# SIRT6 histone deacetylase functions as a potential oncogene in human melanoma

**DOI:** 10.18632/genesandcancer.153

**Published:** 2017-09

**Authors:** Liz Mariely Garcia-Peterson, Mary Ann Ndiaye, Chandra K. Singh, Gagan Chhabra, Wei Huang, Nihal Ahmad

**Affiliations:** ^1^ Department of Dermatology, University of Wisconsin, Madison, Wisconsin, USA; ^2^ Department of Pathology and Laboratory Medicine, University of Wisconsin, Madison, Wisconsin, USA; ^3^ William S. Middleton VA Medical Center, Madison, Wisconsin, USA

**Keywords:** sirtuins, SIRT6, melanoma, autophagy, senescence

## Abstract

Melanoma is an aggressive skin cancer that can rapidly metastasize to become fatal, if not diagnosed early. Despite recent therapeutic advances, management of melanoma remains difficult. Therefore, novel molecular targets and strategies are required to manage this neoplasm. This study was undertaken to determine the role of the sirtuin SIRT6 in melanoma. Employing a panel of human melanoma cells and normal human melanocytes, we found significant SIRT6 mRNA and protein upregulation in melanoma cells. Further, using a tissue microarray coupled with quantitative Vectra analysis, we demonstrated significant SIRT6 overexpression in human melanoma tissues. Lentiviral short hairpin RNA-mediated knockdown of SIRT6 in A375 and Hs 294T human melanoma cells significantly decreased cell growth, viability, and colony formation, induced G1-phase arrest and increased senescence-associated beta-galactosidase staining. As autophagy is important in melanoma and is associated with SIRT6, we used a qPCR array to study SIRT6 knockdown in A375 cells. We found significant modulation in several genes and/or proteins (decreases in AKT1, ATG12, ATG3, ATG7, BAK1, BCL2L1, CLN3, CTSB, CTSS, DRAM2, HSP90AA1, IRGM, NPC1, SQSTM1, TNF, and BECN1; increases in GAA, ATG10). Our data suggests that increased SIRT6 expression may contribute to melanoma development and/or progression, potentially via senescence-and autophagy-related pathways.

## INTRODUCTION

Melanoma is one of the deadliest forms of skin cancer, and can rapidly metastasize to become lethal if not diagnosed early or left untreated. It is estimated that 87,110 new cases of melanoma will be diagnosed in the US alone in 2017 [1]. Current options for melanoma management include surgical resection (if caught early without any sign of distant metastasis), radiation therapy, immunotherapy, and specific pathway targeted drugs such as B-Raf proto-oncogene, serine/threonine kinase (BRAF) inhibitors Vemurafenib (Zelboraf) and dabrafenib (Tafinlar) [2]. However, significant adverse events, as well as tumor resistance and recurrence frequently prevent these treatments from being completely successful [3-6]. Further, even with these advanced treatments, 9,730 deaths are estimated to occur in 2017 in the US due to this neoplasm [1]. Therefore, novel molecular targets and treatments are required for effective management of this cancer. Melanoma provides an ideal neoplasm for new molecularly-targeted drug development, since genetic alterations contribute greatly in the pathogenesis of this disease. In this direction, our laboratory has been interested in identifying the role and functional significance of the sirtuin family of proteins in melanoma [7-13].

The sirtuins (SIRTs) are a family of seven (SIRTs 1-7) NAD-dependent mammalian histone and protein deacetylases that regulate multiple pathways involved in aging, metabolism and cancer. These important enzymes have been found to impact a wide variety of cellular and organismal processes, including, metabolism, transcriptional regulation, DNA repair, and aging [13-16]. Since many of these processes are involved in cancer initiation and progression, sirtuins are considered an attractive target in cancer research. Although research to date indicates that sirtuins are involved in cancer, their role is extremely complex and appears to depend on the cellular context, with many sirtuins showing both tumor suppressor as well as oncogenic properties [17]. Like other sirtuins, nuclear SIRT6 is emerging as a critical player in a variety of cellular functions, including regulation of pathways related to gene transcription, glucose homeostasis, DNA repair and telomere integrity [18-20]. Interestingly, investigations into the exact role of SIRT6 in cancer appears to result in mixed answers, with available evidence for both tumor promoter as well as tumor suppressor roles of this sirtuin [18, 21-25]. However, the role of SIRT6 in melanoma is unknown. This study was undertaken to determine the role and functional significance of SIRT6 in melanoma.

## RESULTS

### SIRT6 is overexpressed in human melanoma cell lines and clinical tissues

**Figure 1:**
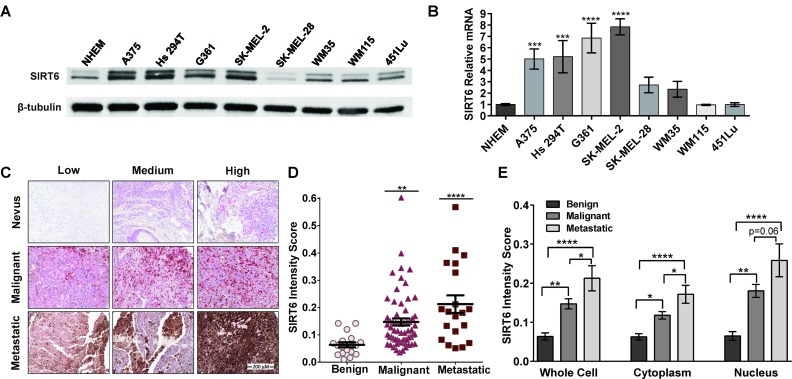
SIRT6 is overexpressed in melanoma cells and clinical tissues (A) Melanoma cells were grown to 80% confluency and cell lysates were prepared. SIRT6 protein levels were determined by western blot analysis. Equal loading was confirmed by reprobing the blot for β-tubulin. (B) The relative expression of the SIRT6 transcript in melanoma cell lines was determined by RT-qPCR using the ΔΔCT comparative method with GAPDH as an endogenous control. The data are shown as mean ±SE of three experiments. (C) A tissue microarray (TMA) containing 100 nevi and melanoma patient tissue samples was analyzed via immunohistochemical staining for SIRT6 protein. Tissues were stained with primary anti-SIRT6 antibody, secondary antibody, and hematoxylin as a counterstain, and representative images are shown (scale bar=200 μm). Individual tissue cores on the TMA were analyzed through Vectra/inForm quantitation. Individual whole cell (D) and mean cellular compartment (E) protein levels are plotted ±SE. Statistical significance denoted as ^*^ P ≤ 0.05, ^**^P≤0.01, ^***^P≤0.001, ^****^P<0.0001.

As the first step of our investigation into understanding the role of SIRT6 in melanoma, we determined the expression profile of SIRT6 in normal human epidermal melanocytes (NHEM) and a panel of human melanoma cell lines (A375, Hs 294T, G361, SK-MEL-2, SK-MEL-28, WM35, WM115 and 451Lu). We found that compared to NHEM, most of the melanoma cell lines exhibited significantly higher expression of SIRT6 protein and mRNA as assessed by immunoblot and RT-qPCR analyses, respectively (Figure 1A-B). Next, to determine the clinical significance of SIRT6 in melanoma, we assessed the expression pattern of SIRT6 in human tissues using a human melanoma tissue microarray (TMA), containing a total of 62 cases of malignant melanoma, 20 metastatic malignant melanoma and 18 nevus tissues. Individual tissue cores were analyzed with Vectra/inForm quantitation. As shown in Figure 1C-E (and detailed in Supplementary Figure 1 and Supplementary Table 1), compared to melanocytic nevi, we found a significantly higher expression of SIRT6 in melanoma. Interestingly, we found SIRT6 overexpression both in the cytoplasm, as well as in nucleus of the cells (Figure 1E). Moreover, metastatic samples were found to have more SIRT6 than primary malignant melanoma samples. This differential expression of SIRT6 in melanoma encouraged us to further explore its role in melanoma.

### SIRT6 knockdown inhibits growth and clonogenic survival of A375 and Hs 294T human melanoma cell lines

**Figure 2:**
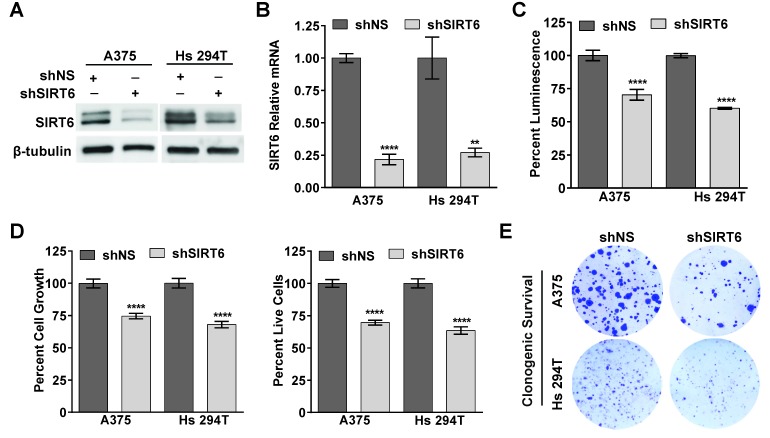
SIRT6 knockdown inhibits proliferation and colony formation in melanoma cells A375 and Hs 294T melanoma cells were transduced with nonsense (NS) and SIRT6 shRNA for 48 h, and cells were analyzed 48 h later. Cell lysates were subjected to (A) western blot and (B) RT-qPCR to confirm SIRT6 knockdown. β-tubulin and GAPDH were used as controls, respectively. (C) Viability of cells upon SIRT6 knockdown was determined by Promega RealTime-Glo MT Cell Viability Assay. (D) Cell growth and viability were also determined by trypan blue exclusion assay. Results are expressed as percentage of viable or total shSIRT6 cells compared to shNS. The data are expressed as mean ±SE of three experiments and statistical significance is denoted as ^**^P≤0.01, ^****^P≤0.0001. (E) To test clonogenic survival, 48 h after transduction an equal number of viable cells were plated into new 6-well plates. After ∼10 days, cells were fixed and stained with crystal violet and scanned. The images shown are representative of three separate experiments with similar results.

As an initial assessment to understand the functional significance of SIRT6 in melanoma, we employed lentiviral-mediated transient transfection of short hairpin RNA (shRNA) to knockdown the SIRT6 gene expression in A375 and Hs 294T cells. To determine the optimal shRNA for knockdown, we tested five different shRNA constructs and chose the one with the greatest knockdown as compared to nonsense shRNA (shNS) (Supplementary Figure S2) for subsequent studies. As shown in Figure 2A-B, we achieved significant knockdown of SIRT6 in both A375 and Hs 294T cells, both at the protein and mRNA levels. Next, we assessed the effects of SIRT6 knockdown on the proliferative potential of melanoma cells using the Promega RealTime-Glo MT Cell Viability Assay (Figure 2C) and the trypan blue exclusion assay (Figure 2D). Our data demonstrated that SIRT6 knockdown significantly decreased cell growth and viability in both of the melanoma cell lines tested. We further assessed the long-term clonogenic survival of the cells following SIRT6 knockdown. As seen in Figure 2E, SIRT6 knockdown resulted in a marked decrease in colony formation abilities of A375 and Hs 294T melanoma cells, suggesting that knockdown of SIRT6 in melanoma cells results in markedly diminished long-term proliferative potential.

### SIRT6 knockdown induces G1-phase arrest and senescence-like phenotypes in human melanoma cells

**Figure 3:**
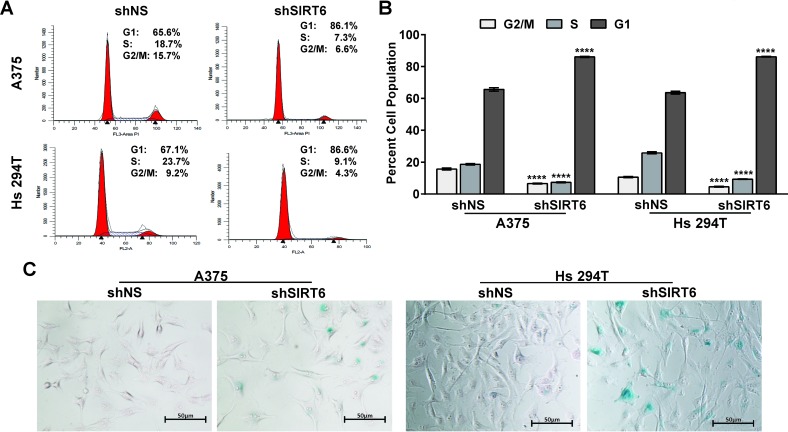
SIRT6 knockdown induces G1-phase arrest and senescence-like phenotypes in human melanoma cells Cell cycle analysis was performed using propidium iodide (PI) staining 48 h post transduction in A375 and Hs 294T cells. Representative histograms and mean cell cycle values from 3 separate experiment are shown (A), as well as mean percent of cells in each phase of the cell cycle (B). Data are shown as mean ±SE with statistical significance denoted as ^****^P≤0.0001. (C) Senescence-associated β-galactosidase staining was performed on A375 and Hs 294T cells 48 h post SIRT6 knockdown, showing accumulation of blue color (senescence-associated vacuoles) in senescent cells. Representative images of three separate experiments with similar results are shown.

To determine if the observed anti-proliferative response of SIRT6 knockdown in melanoma cells was associated with a dysregulation in cell cycle, we performed a cell cycle distribution analysis by flow cytometry (Figure 3A-B). We found that SIRT6 knockdown resulted in an enhanced accumulation of cells in G0/G1 phase in both A375 and Hs 294T human melanoma cell lines. This increase in G1 phase was accompanied by a concomitant decrease of cells in S phase and G2/M phase. The finding of increased G1 arrest, paired with observation of a multinucleated phenotype in our cell cultures after knockdown (data not shown), suggested the possible induction of senescence in the cells. To confirm this, we determined the effects of SIRT6 knockdown on senescence-associated beta-galactosidase (SA-β-Gal) staining in A375 and Hs 294T. As shown in Figure 3C, we found that SIRT6 knockdown resulted in an appreciable increase in SA-β-Gal positive cells, suggesting senescence induction.

### SIRT6 knockdown causes modulation of autophagy-related genes in melanoma cells

**Figure 4:**
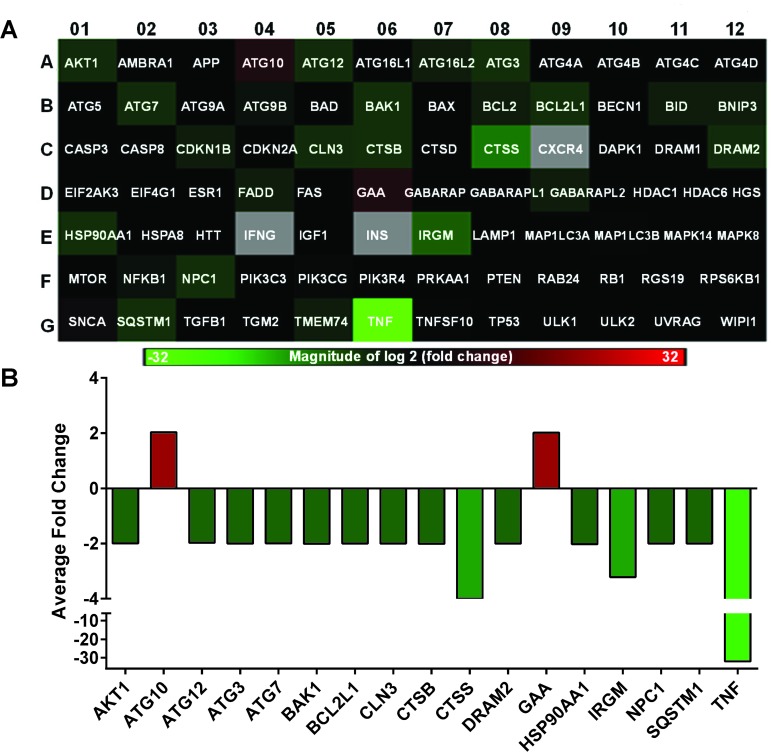
SIRT6 knockdown significantly alters autophagy-related genes in A375 melanoma cells Qiagen Autophagy RT-qPCR array was performed using SIRT6 knockdown A375 cells. The data shown are mean of three biological replicates. (A) A heat map was generated from the data to display fold changes in the tested autophagy-related genes. Upregulated genes are displayed in red and downregulated genes are shown in green, black boxes indicate no/negligible fold change. (B) Graphical representation of the 17 genes that were found to be ≥2 fold differentially expressed in the Autophagy PCR Array.

Because autophagy and senescence are believed to be functionally intertwined processes [26], we next wanted to assess the effects of SIRT6 knockdown on autophagy-related pathways in A375 melanoma cells. To achieve this, we employed a commercially available autophagy PCR array containing 84 genes that are related to autophagy. A number of genes in the array were found to be modulated following SIRT6 knockdown in A375 cells. Detailed information and fold change in genes following SIRT6 knockdown are provided in Supplementary Table 4. A heat map generated from the data obtained is shown in Figure 4A. We found that 17 of the 84 genes tested were significantly modulated by two-fold or more (Figure 4B). Of these 17 genes, 2 were upregulated (*ATG10* and *GAA*) and 15 were downregulated (*AKT1, ATG12, ATG3, ATG7, BAK1, BCL2L1, CLN3, CTSB, CTSS, DRAM2, HSP90AA1, IRGM, NPC1, SQSTM1* and *TNF*). Out of the 17 genes, only *CTSS* and *TNF* were found to have more than 4-fold changes.

### Modulations in autophagy-related genes are associated with the antiproliferative response of SIRT6 inhibition

**Figure 5:**
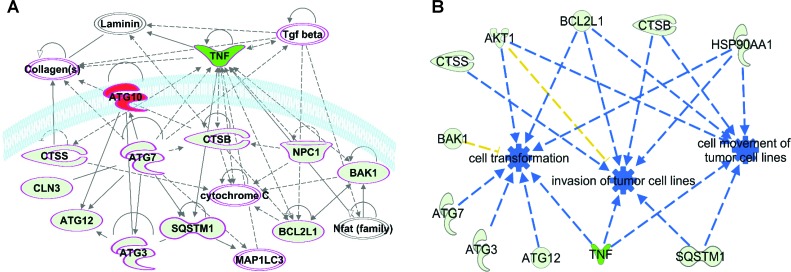
SIRT6 modulated autophagy-related genes appear to be involved in cancer and are predicted to play roles in cell transformation, tumor invasion and movement of tumor cells (A) A network pathway showing regulatory interactions among 12 of the 17 genes modulated ≥2-fold upon SIRT6 knockdown was generated using IPA. Pink boundaries indicate involvement of those genes in cancer. Gene-gene interactions are indicated by arrows, solid lines denote robust correlation with partner genes, and dashed lines indicate statistically significant but less frequent correlations. Upregulated genes after SIRT6 knockdown are represented in red color whereas downregulated are shown in green. Uncolored nodes indicate genes not tested in the RT-qPCR array. (B) IPA was further used to explore the functions of SIRT6-modulated genes. The functional annotations leading to inhibition are denoted with blue dotted lines. Findings inconsistent with the state of downstream molecules are represented with the dark yellow dotted lines.

In order to understand and connect the SIRT6-mediated modulations in autophagy-related genes, the 17 genes that were ≥2-fold modulated were analyzed using IPA and a network pathway was generated that connected 12 of the 17 genes that were found to be modulated in PCR array (Figure 5A). Interestingly, many of the network genes were found to have links with cancer (shown with a pink boundary). IPA was also able to identify links to genes which were not part of the PCR array (denoted as uncolored nodes), suggesting that they may be associated with SIRT6 signaling. IPA was further used to explore the predicted functions of the genes affected by SIRT6 knockdown. It was found that the SIRT6 knockdown-affected genes are associated with cell transformation, tumor invasion and movement of tumor cells (Figure 5B). The highest modulated gene, TNF, appears to affect all three functions predicted by IPA. However, the finding that *BAK1* is downregulated (depicted with yellow lines), appears to be inconsistent with the predicted effect of SIRT6 inhibition.

**Figure 6:**
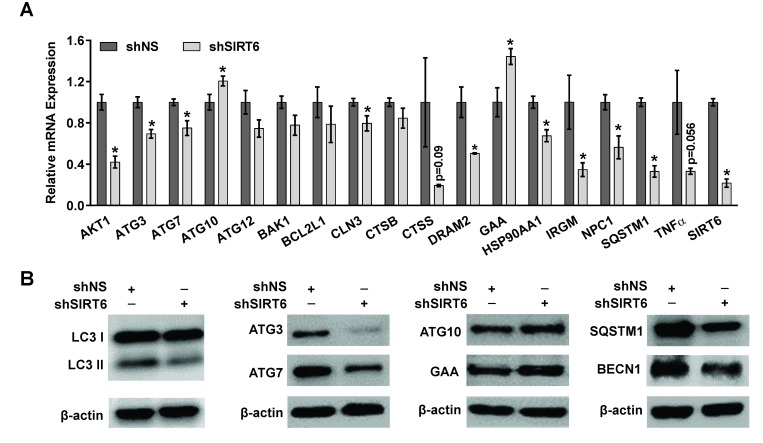
Validation of modulations in autophagy-related markers after SIRT6 knockdown in melanoma cells (A) RT-qPCR analysis was performed to validate the mRNA levels in key altered genes after SIRT6 knockdown A375 cells. Results are expressed as SIRT6 knockdown (shSIRT6) mRNA levels compared to nonsense control (shNS). Data are mean ±SE of three biological replicates, with statistical significance denoted as ^*^P≤ 0.05. (B) Western blot analyses of various autophagy markers including LC3 I and II, BECN1, SQSTM1, ATG3, ATG7, ATG10 and GAA using cell lysates of shNS and shSIRT6 in A375 melanoma cells. β-actin was used as loading control. The figure shows representative western blot images from 2-3 experiments.

To confirm the findings of our PCR array data, we performed RT-qPCR validation of the significantly regulated autophagy-related genes identified above. As shown in Figure 6A, the changes in these 17 genes due to SIRT6 knockdown were comparable to the array data. Further, to determine the modulations in autophagy markers at the protein level, we performed immunoblot analyses using specific antibodies against selected autophagy marker proteins. As shown in Figure 6B, we found significant modulation in various autophagy-related proteins in SIRT6 knockdown cells compared to nonsense control cells. SIRT6 knockdown was found to cause a marked decrease in the protein levels of LC3 II (autophagy related form of LC3), ATG3, ATG7, SQSTM1 and BECN in melanoma cells. However, GAA and ATG10 protein levels were found to be increased, following SIRT6 knockdown in melanoma cells. These results further confirmed that SIRT6 knockdown-mediated anti-proliferative response is associated with modulations in autophagy in melanoma cells.

## DISCUSSION

The objective of this study was to determine the role of the nuclear sirtuin SIRT6 in melanoma. Based on limited available information to date, the role of SIRT6 in cancer is somewhat controversial. While some studies have suggested a tumor suppressor function of SIRT6 in liver and intestinal cancers [18, 21, 22], others have shown that it has oncogenic properties in prostate cancer and non-melanoma skin cancer [23-25]. Thus, the role of SIRT6 in cancer appears to be quite complex and possibly context-and tissue-specific. Interestingly, SIRT6 has been suggested to be an oncogene in keratinocyte-derived skin cancers. Ming *et al* demonstrated that skin-specific SIRT6 deletion inhibited tumorigenesis in mice [25]. Mechanistically, it was found that SIRT6 promoted COX-2 activation via repressing AMPK signaling, thereby increasing cell proliferation and survival in the epidermis [25]. Another finding supporting the pro-proliferative roleof SIRT6 in keratinocytes stems from the proposed inverse association between SIRT6 and the tumor suppressor microRNA34a in keratinocyte differentiation [23]. These findings suggested that SIRT6 could be oncogenic in skin, especially in the keratinocyte-derived cells. Our study focused on melanocytic cells and we found strong evidences supporting the pro-proliferative function of SIRT6 in melanoma.

Our data demonstrated that SIRT6 is upregulated, both at mRNA and protein levels, in melanoma cell lines as well as clinical tissue samples of human melanoma. Thus, our data appears to be consistent with the pro-proliferative function of SIRT6 as seen in prostate and non-melanoma skin cancer [23-25]. In addition, we found that RNA interference-mediated knockdown of SIRT6 reduces cellular growth, viability, and colony formation in A375 and Hs 294T melanoma cells, further underlining the potential pro-proliferative function of SIRT6 in melanoma. Interestingly, our data demonstrated that SIRT6 knockdown induces senescence in melanoma cells as evident by morphological changes (characterized by multinucleated phenotype), as well as increased SA-β-Gal staining [27]. Our data also demonstrated that SIRT6 knockdown produced a marked accumulation of cells in G1 phase at the expense of G2/M and S phases. Senescent cells are generally believed to arrest in G1 or G0 stage of the cell cycle, thus indicating the possibility that SIRT6 knockdown causing the cells to become senescent. This is quite similar to the results we have reported earlier for SIRT3 [7].

In an effort to uncover the mechanism behind the anti-proliferative effects shown by the SIRT6 knockdown in melanoma we performed an autophagy PCR array. Autophagy is an extremely orchestrated process involving activities of various autophagy-related proteins known as ATGs. Recent studies have implicated SIRT6 in senescence [28-30] as well as autophagy [31, 32]. Indeed, autophagy and senescence are believed to have multiple and often overlapping and complementary functions in cancer, both in terms of influencing tumor development and in modulating the response to chemotherapy and/or radiation [33]. Autophagy regulatory proteins work by activating autophagy and beclin 1 regulator 1 (AMBRA1), ATG8/microtubule-associated light chain 3 (LC3), and Sequestosome1 (SQSTM1), followed by the inactivation of mammalian target of rapamycin (mTOR) to induce activation [34]. Interestingly, autophagy has been shown to suppress tumorigenesis by degrading oncogenic and toxic proteins [35, 36] or promote tumor development by reducing cancer cell vulnerability to stress and maintaining tumor cells homeostasis [37]. Our results demonstrate that SIRT6 knockdown significantly alters genes and pathways related to autophagy in melanoma cells both at mRNA and protein levels. Upon SIRT6 knockdown, modulations in various autophagy markers including BECN1, SQSTM1, ATG3, ATG7, ATG10 and GAA were confirmed by PCR array, RT-qPCR and/or immunoblotting. Moreover, SIRT6 knockdown reduced the conversion of LC3 protein from its free form LC3-I to phosphatidylethanolamine-conjugated form LC3-II, which represents an inhibition of a crucial step in autophagosome formation [38]. These data suggest that inhibition of SIRT6 by shRNA inhibits autophagy in melanoma cells. Indeed, SIRT6 has been previously suggested to affect the autophagy in human bronchial epithelial cells and esophageal cancer cells [31, 39, 40]. Based on our data, it appears that SIRT6 positively regulates autophagy in melanoma cells. This is an interesting observation because melanoma researchers seem to have come to a consensus that autophagy is tumor-suppressive in the early stages of cancer and tumor-promoting in established tumors (discussed in [41]). Indeed, future studies are required to carefully study the role of SIRT6 and autophagy in early versus late melanomas.

Overall, our study provides strong evidence suggesting a tumor promoter function of SIRT6 in melanoma, warranting future detailed investigations into the role, mechanism and therapeutic targetability of SIRT6 in melanoma. *In vivo* studies in relevant melanoma models are needed to further validate our *in vitro* findings.

## MATERIALS AND METHODS

### Cell culture

All cell lines are routinely (upon thawing, or if a cell line is in culture for more than 3 months) authenticated using STR testing through our core facility, the University of Wisconsin Translational Research Initiatives in Pathology (TRIP) lab. For our experiments, human melanoma cell lines (A375, Hs 294T, G361, SK-MEL-2, SK-MEL-28, WM35, WM115, 451Lu) and normal adult human melanocyte (NHEM) were purchased from ATCC, and HEK293T Lenti-X cells were purchased from TaKaRa Bio. SK-MEL-28, WM35 and SK-MEL-2 were maintained in EMEM supplemented with 1 mM sodium pyruvate and non-essential amino acids, G361 was maintained in McCoy's 5a medium (Corning) and HEK293T Lenti-X, Mel-ST, A375, Hs 294T were maintained in DMEM (Corning), all with 10% fetal bovine serum (Sigma-Aldrich) and NHEMs were cultured in dermal cell medium supplemented with adult growth supplement (ATCC). All the cells were maintained at standard cell culture conditions (37°C, 5% CO_2_ in humidified incubator).

### Reverse transcription-quantitative real-time PCR (RT-qPCR)

For RT-qPCR analysis, total RNA was isolated from cells using the RNeasy Mini Kit (Qiagen), and first strand cDNA was transcribed using Oligo(dT) primers, dNTPs and M-MLV reverse transcriptase (Promega). RT-qPCR was perform using the BioRad CFX96 Touch and the BioRad iTaq Universal SYBR Green Supermix with first strand cDNA, forward and reverse gene-specific primer pairs. Melt curve analysis was performed to ensure the specificity of target amplicon. Relative mRNA levels were analyzed using *GAPDH* as endogenous control and ΔΔCT algorithm using the BioRad CFX Manager software.

### Immunohistochemical (IHC) staining

Human melanoma TMA was purchased from US Biomax (Cat #ME1004c), and was stained as described previously [7]. Briefly, slides were deparaffinized in xylene, and rehydrated through a standard graded ethanol series. Antigen retrieval was performed using IHC-Tek epitope retrieval solution and steamer set (IHC World, LLC.). The slides were then immersed in 3% H_2_O_2_ for endogenous peroxidase quenching. After blocking with goat serum, Vectastain ABC-AP Kit and Vector Red Alkaline Phosphatase Substrate Kit I (Vector Labs) were used for tissue staining as per manufacturer protocol. SIRT6 primary antibody (Cell Signaling, Cat#12486) was used at 1:150 dilution. The slides were then counterstained with Harris modified hematoxylin (ThermoFisher Scientific Inc.), dehydrated with graded ethanol and xylenes, and mounted with Permount before undergoing image analysis with the Vectra system, as described below.

### TMA image acquisition and analysis

For quantitative staining analysis, the Vectra automated quantitative tissue imaging system, including an automated slide scanner and state-of-the-art software (Nuance and inForm; PerkinElmer) was used, as described previously [8]. This system takes advantage of the unique spectral characteristics (curve) of each chromogen via creation of a spectral library from single-chromogen slides in order to determine the relative contribution of each chromogen to the stained area on a slide. The stained TMA was imaged with the Vectra slide scanner, using a scanning protocol that was created based on the core size and layout, as well as acquisition of the spectral library. For each slide, an 8-bit image cube from each of the TMA tissue cores was acquired and inForm software was used to segment tissues (melanoma *versus* others) and cells (nucleus *versus* cytoplasm) to analyze the protein levels. Then, the target SIRT6 signals (Vector Red staining) were quantitated within the tissue and subcellular compartment(s) after unmixing the spectral curves with inForm software. Using these systems allowed for elimination of signal noises and cross-talk. Continuous signal intensity data (mean optical density per pixel) was generated for each TMA core or cellular compartment.

### Lentiviral production and transduction

For viral creation, Lenti-X 293T cells were passaged to attain 30–40% confluency the next day and then transfected using the CaPO_4_ method, as described previously [42]. Five SIRT6-targeting shRNA constructs were purchased and the clone with the best knockdown was chosen for further study. Briefly, 8 μg shRNA plasmid DNA (nonsense or SIRT6-targeting shRNA) (Sigma Aldrich, sequence information in Supplementary Table S2), 0.5 μg of VSV-G, and 0.6 μg of Δ8.2 plasmids were mixed with sterile ddH_2_O and CaCl_2_. The DNA mix was bubbled, and an equal volume of 2x HEPES buffered saline (pH 7.05) was added dropwise. The transfection mixture was added to the appropriate dishes and incubated overnight. After 24 h, the transfection media was removed and fresh media was added. After an additional 24 and 48 h, cell media containing shRNA lentivirus was collected and filtered before use. For target cell transduction, 1.5-2.0 × 10^4^ melanoma cells (A375 and Hs 294T) were plated in each well of a 6-well plate. Viral media was added to triplicate sets of cells along with 8 μg/ml of polybrene a total of four times over 2 days. After 48 hours of transduction, the viral media was removed and cells were allowed to recover for 48 hours before being collected for further experiments.

### Trypan blue exclusion and RealTime-Glo MT cell viability assays

For trypan blue exclusion assay, following treatments and recovery time as outlined above, the cells were trypsinized, an aliquot was stained with trypan blue dye (ThermoFisher Scientific), and the viable, dead, and total cells were determined using the Countess II FL Automated Cell Counter (ThermoFisher Scientific). To further confirm the effects of SIRT6 knockdown, we used RealTime-Glo MT cell viability assay (Promega), according to the manufacturer's protocol. For this purpose, following treatments, 500 cells were plated per well in 96 well plates and transduced as above. Twenty-four hours after transduction, RealTime-Glo reagent was added to the wells, and luminescence was measured at 48 h using the BioTek Synergy H1 plate reader.

### Clonogenic cell survival assay

Forty eight hours after transductions, 200 viable cells from each treatment group were plated in separate wells of a 6-well plate. Cells were maintained under standard tissue culture conditions, changing media every 3 days for approximately 10 days, until the cells reached optimal density. Cells were then stained at room temp for 30 m with 0.5% crystal violet solution in 1:1 methanol:water, rinsed 2 times with phosphate buffered saline (pH 7.4), and air dried followed by digital photography.

### Senescence-associated β-galactosidase (SA-β-Gal) staining

Senescence-associated beta-galactosidase catalyzes the hydrolysis of β-galactosides into monosaccharides in senescent cells. To determine the induction of senescence, 1000 cells were plated per well in 6 well plates and transduced as above. Forty eight hours after transduction, cells were stained using the Senescence β-Galactosidase Staining Kit (Cell Signaling) per manufacturer's instructions. The next day, the cells were washed with PBS and counterstained with Nuclear Fast Red (Vector Laboratories) for 30 sec. The images of SA-β-gal positive cells (with blue color sedimentation in cytoplasm) were captured with a Nikon DS-Fi1 microscope (Nikon Instruments Inc.) using NIS Elements AR 3.1 software.

### Cell cycle analysis

To determine the impact of SIRT6 knockdown on cell cycle, 15000-18000 cells were plated per well in 6-well plates and transduced as above. Forty-eight hours after transduction, cells were trypsinized, washed twice with PBS and fixed in ice-cold 70% ethanol overnight. Cells were then pelleted, washed twice with PBS, treated with RNase, and stained with propidium iodide (Invitrogen). Cell cycle distribution was analyzed on a FACScan benchtop cytometer at the University of Wisconsin Paul P. Carbone Comprehensive Cancer Center (UWCCC) Flow cytometry facility. Cell cycle distribution was analyzed using CellQuest software (BD Biosciences). Cell cycle analysis was performed using ModFit software (Verity Software House)

### Immunoblot analysis

Protein lysates were prepared, quantified and subjected to SDS-PAGE followed by transferring to nitrocellulose membranes. The proteins were probed using primary antibodies (SIRT6, #12486; β-tubulin, #2128; ATG3, #3415; ATG7, #8558; Beclin1, #3495; β-Actin, #4970 from Cell Signaling Technologies, LC3B, #NB600-1384 from Novus Biologicals and ATG10, #A9356; GAA, #G2048 from Sigma-Aldrich) and appropriate secondary antibodies (anti-rabbit, #7404; anti-mouse, #7076, Cell Signaling Technologies) followed by chemiluminescence detection with Pierce ECL Western Blotting Substrate (Thermo Scientific). Blots were imaged using the ImageQuant LAS 4000 (GE Healthcare).

### Autophagy RT2 profiler PCR array analysis

RT^2^ Profiler™ PCR Arrays for Human Autophagy were purchased from Qiagen (Cat. #PAHS-084Z) and were used and analyzed according to the manufacturer's instructions, employing the TaKaRa SYBR Premix ExTaq II polymerase. Five reference genes (*B2M, HPRT1, RPLP0, GAPDH*, and *ACTB*) were used for normalizing data between shNS-A375 cells and shSIRT6-A375 cells. Data for PCR array were analyzed using SABiosciences RT2 Profiler PCR Data Analysis software and were considered significant at ≥2 fold change and P<0.05. Further analyses of differentially expressed genes were performed using Ingenuity Pathway Analysis (IPA). The predicted gene-gene interaction networks and functions network were generated and analyzed using inputs of gene identifiers and fold-changes (shNS *versus* shSIRT6). Further, the PCR array data were validated using RT-qPCR analyses as detailed in section 2.2. Primer pairs for *AKT1, ATG10, ATG12, ATG3, ATG7, BAK1, BCL2L1, CLN3, CTSB, CTSS, DRAM2, GAA, HSP90AA1, IRGM, NPC1, SQSTM1* and *TNF* were retrieved from PrimerBank [43] and are detailed in Supplementary Table 3.

### Statistical Analysis

All quantitative data were analyzed using GraphPad PRISM 5.0 software (GraphPad Software, Inc., La Jolla, CA). For TMA, statistical significance was determined by one-way ANOVA with Tukey's post hoc test or by two-tailed Student's t-test. For all other quantitative data, statistical analyses were performed with two-tailed unpaired Student's t-test between two experimental groups and one-way analysis of variance for more than two experimental groups followed by Sidak post hoc test. Data were shown as mean ±SE unless otherwise specified. A p-value < 0.05 was considered to be statistically significant.

## SUPPLEMENTARY FIGURES AND TABLES



## ABBREVIATIONS

AKT1: AKT serine/threonine kinase 1
AMPK: Activated Protein kinase
ATG10: autophagy related 10
ATG12: Autophagy related 12
ATG3: Autophagy related 3
ATG7: Autophagy related 7
BAK1: BCL2 antagonist/killer 1
BCL2L1: BCL2 like 1
BRAF: B-Raf proto-oncogene, serine/threonine kinase
CLN3: CLN3, battenin
COX-2: Cyclooxygenase-endoperoxide synthase 2
CTSS: Cathepsin S
CTSB: Cathepsin B
DRAM2: DNA damage regulated autophagy modulator 2
GAA: Glucosidase alpha, acid
GAPDH: Glyceraldehyde-3-phosphate dehydrogenase
HSP90AA1: Heat shock protein 90 alpha family class A member 1
IRGM: Immunity related GTPase M
LC3: Microtubule-associated protein 1A/1B-light chain 3
NHEM: Normal human epidermal melanocytes
NPC1: NPC intracellular cholesterol transporter 1
PI: Propidium iodide
RT-qPCR: Reverse transcription, real-time quantitative polymerase chain reaction
SA-β-Gal: Senescence-associated betagalactosidase
shRNA: Short hairpin RNA
SIRT: Sirtuin
SQSTM1: Sequestosome 1
TMA: Tissue microarray
TNF: Tumor necrosis factor
