# Supplementary material for: SIRT6 histone deacetylase functions as a potential oncogene in human melanoma
Source: Genes Cancer. 2017 Sep;8(9-10):701–12. doi: 10.18632/genesandcancer.153 (PMC5724804; doi:10.18632/genesandcancer.153)
Supplement: Supplementary file 1 [file ganc-08-701-s001.pdf]

SIRT6 histone deacetylase functions as a potential oncogene in human melanoma -

Garcia-Peterson et al

Supplementary Figure S1A

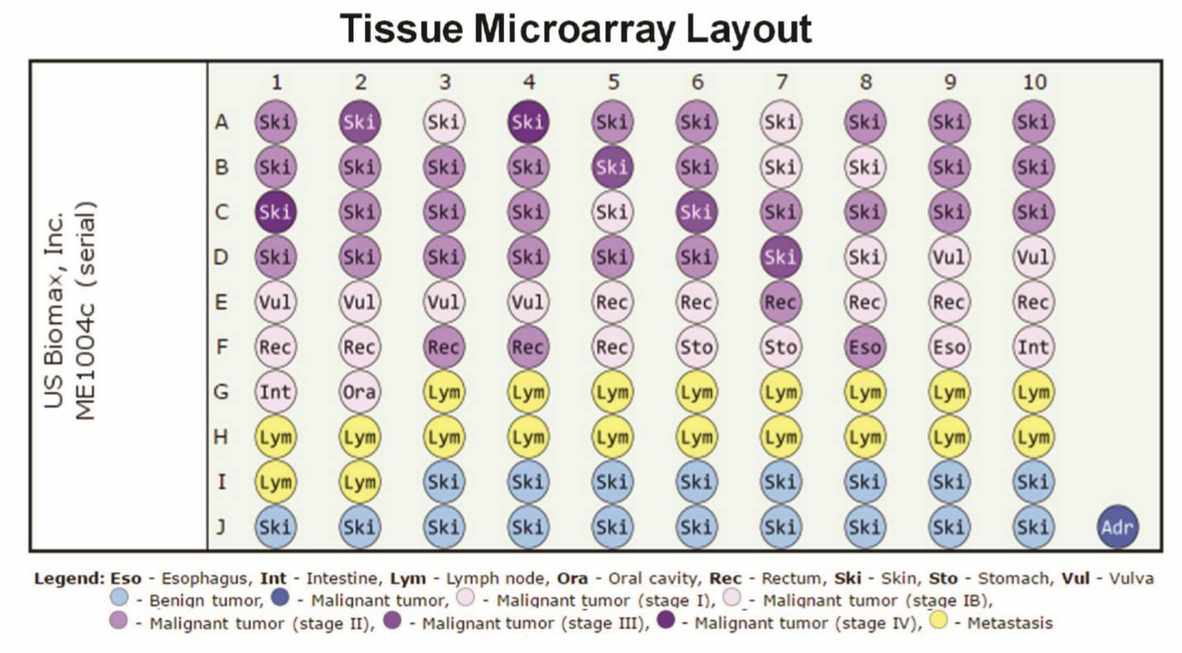

Supplementary Figure S1B

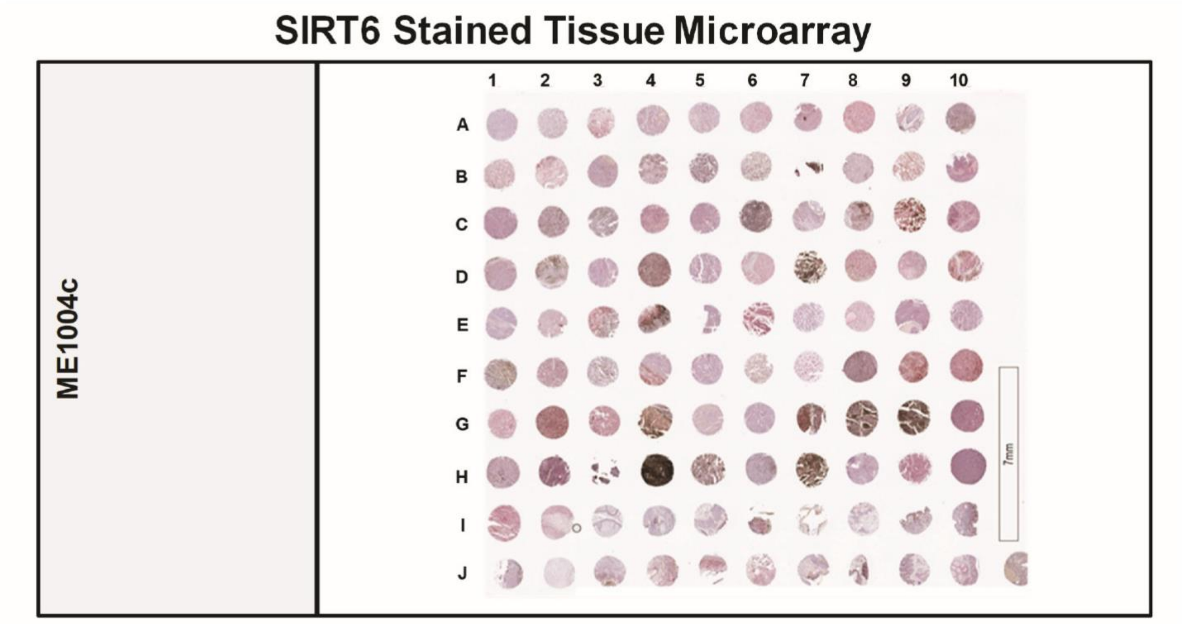

Supplementary Figure S1:Tissue microarray (TMA) staining and organization. A) Diagram of the TMA indicating location and tissue type of each core. B) Whole image of the SIRT6-stained slide with hematoxylin counterstaining. ME1004c indicates the catalog number from US Biomax, Inc that was used for the study.

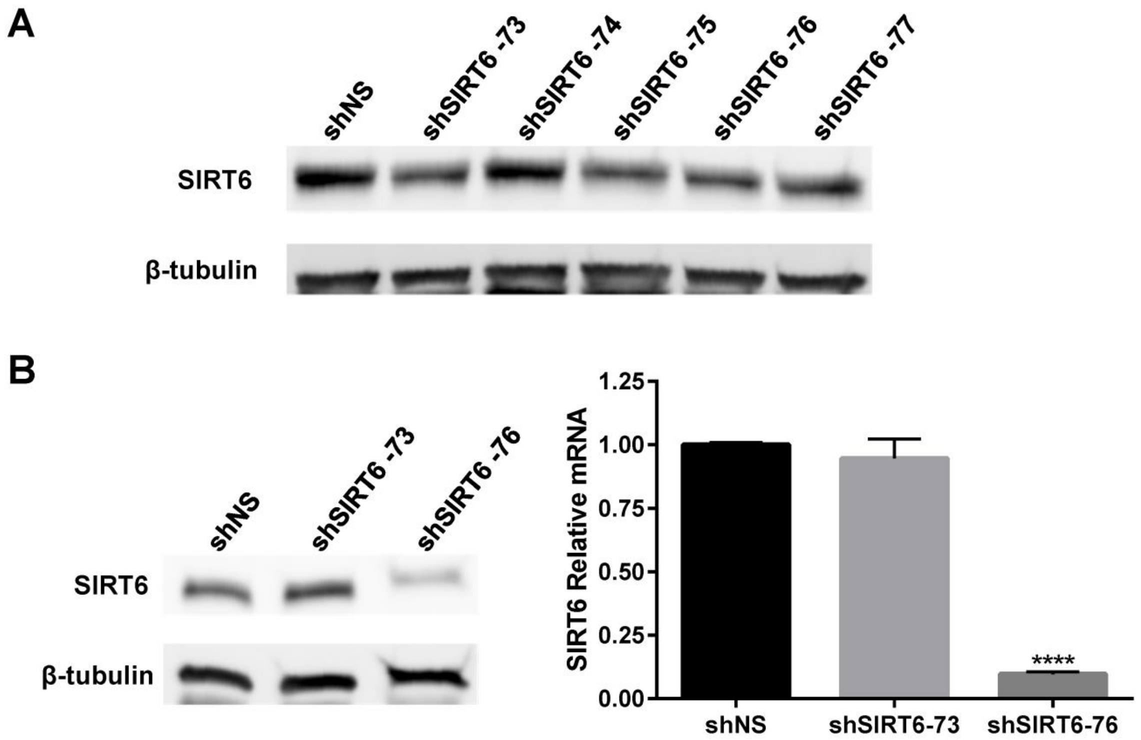

Supplementary Figure S2: Multiple SIRT6-targeting shRNA constructs were analyzed for SIRT6 knockdown. (A) A375 melanoma cells were transfected using Lipofectamine 2000 using manufacturer's protocol (using 4 µg of DNA and 10 µl of Lipofectamine reagent per well in six well plate) with nonsense (NS) and five SIRT6-targeting shRNA for 48 h, and cells were analyzed 48 h later. Cell lysates were subjected to SDS-PAGE and western blotting for SIRT6 protein as in Materials and Methods. Equal loading was confirmed by reprobing the blot for β-tubulin. Further, the two best constructs were chosen to determine which had the best knockdown via lentiviral transduction. (B) A375 cells were transduced with nonsense (NS) and the two best SIRT6 knockdown constructs from A, -73 and -76, for 48 h and collected 48 h later. Cell lysates were subjected to western blot and RT-qPCR to confirm SIRT6 knockdown. β-tubulin and GAPDH were used as controls, respectively.

Supplementary Table S1: Quantification of SIRT6 staining and description of samples.

Type	Sex	Age	Organ	Pathology	Stage	TNM	Position	Nuclear SIRT6 Mean	Cytoplasmic SIRT6 Mean	Whole Cell SIRT6 Mean
Malignant	M	42	Adrenal gland	Pheochromocytoma (tissue marker)	–	–	–	–	–	–
Benign	F	2	Skin	Intradermal nevus of right leg	–	–	J2	0.005	0.015	0.01
Benign	M	38	Skin	Compound nevus of right face	–	–	I3	0.012	0.02	0.016
Benign	M	25	Skin	Compound nevus of left leg	–	–	I8	0.02	0.032	0.027
Benign	F	29	Skin	Compound nevus of left shoulder	–	–	I4	0.022	0.035	0.029
Benign	F	30	Skin	Intradermal nevus of left rump	–	–	I5	0.024	0.041	0.033
Benign	M	20	Skin	Compound nevus of left shoulder	–	–	J1	0.03	0.044	0.038
Benign	M	39	Skin	Intradermal nevus of head	–	–	J7	0.04	0.049	0.045
Benign	F	34	Skin	Sebaceous nevus of head (skin tissue)	–	–	J9	0.05	0.061	0.056
Benign	F	23	Skin	Compound nevus of right lumbar part	–	–	I7	0.073	0.043	0.058
Benign	F	2	Skin	Junctional nevus of neck	–	–	I9	0.057	0.065	0.061
Benign	M	50	Skin	Compound nevus of upper arm	–	–	J3	0.065	0.061	0.062
Benign	M	18	Skin	Sebaceous nevus of left frontal region (skin tissue)	–	–	J10	0.069	0.07	0.069
Benign	M	6 mo	Skin	Intradermal nevus of face	–	–	I10	0.067	0.076	0.072
Benign	M	11	Skin	Intradermal nevus of left thigh	–	–	J8	0.088	0.069	0.078
Benign	M	42	Skin	Intradermal nevus of face	–	–	J4	0.097	0.08	0.087
Benign	M	73	Skin	Compound nevus of left heel	–	–	J6	0.145	0.101	0.12
Benign	F	10	Skin	Junctional nevus of right foot	–	–	I6	0.153	0.133	0.142
Benign	M	55	Skin	Compound nevus of back	–	–	J5	0.152	0.136	0.142
Malignant	M	71	Skin	Malignant melanoma of right rump	II	T4N0M0	A1	0.032	0.04	0.036
Malignant	F	38	Vulva	Malignant melanoma	I	–	E1	0.041	0.043	0.042
Malignant	M	60	Skin	Malignant melanoma of right rump	III	T4N1M0	A2	0.042	0.046	0.044
Malignant	F	41	Skin	Malignant melanoma of scalp	II	T4N0M0	C7	0.056	0.052	0.054
Malignant	F	32	Skin	Malignant melanoma of right lumbar part (fibrofatty tissue)	II	T4N0M0	A9	0.048	0.061	0.055
Malignant	M	79	Skin	Malignant melanoma of left face	I	T1N0M0	B8	0.069	0.059	0.063
Malignant	F	38	Rectum	Malignant melanoma	I	–	E5	0.06	0.065	0.063
Malignant	M	55	Skin	Malignant melanoma of left sole	II	T4N0M0	A5	0.072	0.06	0.065
Malignant	M	64	Rectum	Malignant melanoma	II	–	E7	0.071	0.06	0.065
Malignant	F	46	Skin	Malignant melanoma of right thumb	II	T4N0M0	D5	0.064	0.07	0.067
Malignant	M	55	Stomach	Malignant melanoma	I	–	F6	0.071	0.065	0.067
Malignant	M	61	Skin	Malignant melanoma of left armpit	IV	T4bN0M1	A4	0.061	0.073	0.068
Malignant	M	31	Skin	Malignant melanoma of scalp	II	T4N0M0	D3	0.071	0.072	0.072
Malignant	M	65	Skin	Malignant melanoma of scalp	II	T4N0M0	D1	0.081	0.075	0.078
Malignant	F	44	Vulva	Malignant melanoma	I	–	E2	0.102	0.066	0.081
Malignant	M	41	Skin	Malignant melanoma of left leg	II	T3N0M0	B3	0.091	0.076	0.082
Malignant	M	49	Skin	Malignant melanoma of left arm	II	T4N0M0	C3	0.112	0.055	0.083
Malignant	F	72	Rectum	Malignant melanoma	I	–	F5	0.097	0.07	0.083
Malignant	M	55	Stomach	Malignant melanoma	I	–	F7	0.098	0.068	0.083
Malignant	F	52	Rectum	Malignant melanoma	I	–	E8	0.106	0.07	0.087
Malignant	F	47	Skin	Malignant melanoma of left upper arm	II	T4N0M0	D6	0.101	0.082	0.091
Malignant	M	51	Skin	Malignant melanoma of back	II	T4N0M0	A6	0.104	0.084	0.092
Malignant	F	84	Rectum	Malignant melanoma of crissum	I	–	E9	0.096	0.088	0.092
Malignant	F	67	Rectum	Malignant melanoma	I	–	E10	0.097	0.09	0.093
Malignant	F	42	Skin	Malignant melanoma of right thigh	II	T4N0M0	B1	0.11	0.093	0.101
Malignant	M	75	Rectum	Malignant melanoma	II	–	F3	0.132	0.075	0.101
Malignant	F	72	Vulva	Malignant melanoma	I	–	D9	0.115	0.093	0.103
Malignant	M	50	Skin	Malignant melanoma of left shoulder (fibrous tissue and blood vessel)	II	T4N0M0	B2	0.139	0.083	0.107
Malignant	F	83	Skin	Malignant melanoma of right little finger	I	T2N0M0	C5	0.133	0.087	0.107
Malignant	M	61	Skin	Malignant melanoma of right groin	III	T4N2M0	B5	0.135	0.098	0.114
Malignant	M	25	Skin	Malignant melanoma of left sole	I	T2N0M0	A7	0.142	0.096	0.115
Malignant	F	66	Rectum	Malignant melanoma	I	–	F2	0.136	0.101	0.116
Malignant	M	42	Skin	Malignant melanoma of left heel	IV	T3N2M1	C1	0.142	0.101	0.118
Malignant	M	80	Skin	Malignant melanoma of right sole	II	T4N0M0	A10	0.154	0.106	0.128
Malignant	M	37	Skin	Malignant melanoma of right upper arm	II	T4N0M0	B4	0.18	0.099	0.135
Malignant	M	72	Skin	Malignant melanoma of left sole	IB	T2aN0M0	A3	0.179	0.108	0.138
Malignant	M	52	Skin	Malignant melanoma of left abdominal wall	II	T4N0M0	B6	0.197	0.081	0.14
Malignant	M	50	Esophagus	Malignant melanoma	II	–	F8	0.162	0.126	0.143
Malignant	M	73	Intestine	Malignant melanoma	I	–	G1	0.178	0.114	0.144

Continued from previous page

Malignant	M	41	Skin	Malignant melanoma of left forearm	II	T4N0M0	C2	0.212	0.096	0.145
Malignant	M	71	Skin	Malignant melanoma of right groin	II	T4N0M0	C4	0.181	0.116	0.147
Malignant	F	54	Rectum	Malignant melanoma of anal tube	II	–	F4	0.17	0.129	0.147
Malignant	M	66	Rectum	Malignant melanoma	I	–	F1	0.21	0.096	0.148
Malignant	M	45	Skin	Malignant melanoma of crissum	II	T4N0M0	D2	0.205	0.103	0.152
Malignant	M	66	Skin	Malignant melanoma of right thigh	II	T4N0M0	C10	0.187	0.141	0.16
Malignant	F	59	Skin	Malignant melanoma with necrosis of anus	II	T4N0M0	B10	0.213	0.123	0.163
Malignant	M	51	Skin	Malignant melanoma of left armpit	II	T4N0M0	C8	0.22	0.127	0.169
Malignant	M	51	Skin	Malignant melanoma of left upper arm	III	T4N1M0	C6	0.222	0.128	0.174
Malignant	F	46	Skin	Malignant melanoma of thigh	II	T4N0M0	A8	0.215	0.17	0.19
Malignant	F	62	Vulva	Malignant melanoma	I	–	E3	0.296	0.145	0.215
Malignant	F	45	Skin	Malignant melanoma of left thigh	I	T2N0M0	D8	0.294	0.159	0.222
Malignant	F	57	Vulva	Malignant melanoma	I	–	D10	0.301	0.17	0.233
Malignant	F	72	Skin	Malignant melanoma of right cheek	II	T4N0M0	D4	0.312	0.17	0.234
Malignant	M	40	Skin	Malignant melanoma of right chest wall	II	T4N0M0	B9	0.293	0.165	0.238
Malignant	F	44	Rectum	Malignant melanoma	I	–	E6	0.356	0.193	0.268
Malignant	M	71	Intestine	Malignant melanoma	I	–	F10	0.329	0.257	0.29
Malignant	F	45	Vulva	Malignant melanoma	I	–	E4	0.422	0.205	0.309
Malignant	M	64	Esophagus	Malignant melanoma	I	–	F9	0.406	0.265	0.328
Malignant	F	59	Skin	Malignant melanoma of right rump	I	T1N0M0	B7	0.43	0.258	0.34
Malignant	F	70	Oral cavity	Malignant melanoma of left parotid gland	I	–	G2	0.412	0.285	0.345
Malignant	M	49	Skin	Malignant melanoma of left foot	III	T4N1M0	D7	0.511	0.278	0.399
Malignant	M	56	Skin	Malignant melanoma of abdominal wall	II	T4N0M0	C9	0.719	0.477	0.603
Metastasis	F	61	Lymph node	Metastatic malignant melanoma from groin	–	–	H6	0.05	0.053	0.051
Metastasis	M	44	Lymph node	Malignant malignant melanoma from right armpit	–	–	G6	0.051	0.057	0.054
Metastasis	F	55	Lymph node	Metastatic malignant melanoma from left groin	–	–	G5	0.061	0.063	0.062
Metastasis	F	41	Lymph node	Metastatic malignant melanoma from right groin (fibrous tissue and blood vessel)	–	–	I2	0.073	0.067	0.07
Metastasis	F	43	Lymph node	Metastatic malignant melanoma from thigh	–	–	H8	0.093	0.073	0.082
Metastasis	M	70	Lymph node	Malignant malignant melanoma from right armpit	–	–	H1	0.145	0.089	0.113
Metastasis	F	56	Lymph node	Malignan tmalignant melanoma from left sole	–	–	H10	0.138	0.128	0.133
Metastasis	M	68	Lymph node	Metastatic malignant melanoma from left groin	–	–	H3	0.158	0.115	0.135
Metastasis	F	41	Lymph node	Metastatic malignant melanoma from right groin	–	–	H5	0.221	0.125	0.168
Metastasis	M	73	Lymph node	Metastatic malignant melanoma from right groin	–	–	H9	0.199	0.166	0.181
Metastasis	F	63	Lymph node	Metastatic malignant melanoma from right heel	–	–	G3	0.213	0.164	0.185
Metastasis	F	40	Lymph node	Metastatic malignant melanoma from right groin	–	–	G10	0.225	0.168	0.194
Metastasis	F	49	Lymph node	Metastatic malignant melanoma from neck	–	–	I1	0.223	0.172	0.194
Metastasis	M	56	Lymph node	Metastatic malignant melanoma from left ear	–	–	H2	0.243	0.187	0.212
Metastasis	F	47	Lymph node	Metastatic malignant melanoma from left thump	–	–	G8	0.426	0.235	0.328
Metastasis	M	72	Lymph node	Metastatic malignant melanoma from right groin	–	–	G7	0.472	0.271	0.362
Metastasis	M	58	Lymph node	Metastatic malignant melanoma from left groin	–	–	G4	0.465	0.278	0.364
Metastasis	F	38	Lymph node	Metastatic malignant melanoma from right leg	–	–	H7	0.49	0.302	0.392
Metastasis	M	63	Lymph node	Metastatic malignant melanoma from left Lower gum	–	–	G9	0.536	0.282	0.41
Metastasis	F	72	Lymph node	Metastatic malignant melanoma from left groin	–	–	H4	0.69	0.439	0.568

"-" indicates fields where data was not available or not provided. Samples without a staining intensity were eliminated because of either lack of sufficient melanocytes or poor section quality for tissue segmentation.

Supplementary Table S2: Details of shRNA vectors used

Vector ID	Clone ID	Sequence
Control Vector (shNS)	N/A	CCGGCAACAAGATGAAGAGCACCAACTCGAGTTGGTGCTCTTCATCTTGTTGTTTTT
TRCN0000050473 (shSIRT6-73)	NM_016539.1-473s1c1	CCGGTGGAAGAATGTGCCAAGTGTACTCGAGTACACTTGGCACATTCTTCCATTTTTG
TRCN0000050474 (shSIRT6-74)	NM_016539.1-186s1c1	CCGGGCAGTCTTCCAGTGTGGTGTTCTCGAGAACACCACACTGGAAGACTGCTTTTTG
TRCN0000050475 (shSIRT6-75)	NM_016539.1-696s1c1	CCGGGCTGGGTACATCGCTGCAGATCTCGAGATCTGCAGCGATGTACCCAGCTTTTTG
TRCN0000050476 (shSIRT6-76)	NM_016539.1-972s1c1	CCGGCACCCGGATCAACGGCTCTATCTCGAGATAGAGCCGTTGATCCGGGTGTTTTTG
TRCN0000050477 (shSIRT6-77)	NM_016539.1-457s1c1	CCGGCACGGGAACATGTTTGTGGAACTCGAGTTCCACAAACATGTTCCCGTGTTTTTG

Supplementary Table S3: Primer sequences used for RT-qPCR validation.

Gene	Amplicon size (bp)	Primer orientation	Primer Sequence (5' - > 3')	Length (bp)	Tm (°C)	Primer Bank ID
SIRT6	194	F	CCCACGGAGTCTGGACCAT	19	63	300797596c1
		R	CTCTGCCAGTTTGTCCCTG	19	60	
AKT1	96	F	AGCGACGTGGCTATTGTGAAG	21	63	62241012c1
		R	GCCATCATTCTTGAGGAGGAAGT	23	62	
ATG10	180	F	ATAAGATGCGACTGCTACAGGG	22	62	196162716c3
		R	CAATGCTCAGCCATGATGTGAT	22	61	
ATG12	153	F	TAGAGCGAACACGAACCATCC	21	62	290560745c2
		R	CACTGCCAAAACACTCATAGAGA	23	60	
ATG3	125	F	GATGGCGGATGGGTAGATACA	21	61	34147490c3
		R	TCTTCACATAGTGCTGAGCAATC	23	60	
ATG7	114	F	ATGATCCCTGTAACTTAGCCCA	22	60	222144228c3
		R	CACGGAAGCAAACAACTTCAAC	22	61	
BAK1	192	F	CATCAACCGACGCTATGACTC	21	61	109698605c3
		R	GTCAGGCCATGCTGGTAGAC	20	62	
BCL2L1	179	F	GACTGAATCGGAGATGGAGACC	22	62	20336333c2
		R	GCAGTTCAAACTCGTCGCCT	20	63	
CLN3	124	F	CGCCCACGACATCCTTAGC	19	63	109698602c1
		R	AGCAGCCGTAGAGACAGAGTT	21	63	
CTSB	159	F	AGAGTTATGTTTACCGAGGACCT	23	60	66346650c3
		R	GATGCAGATCCGGTCAGAGA	20	61	
CTSS	113	F	TGACAACGGCTTTCCAGTACA	21	62	315075310c2
		R	GGCAGCACGATATTTTGAGTCAT	23	61	
DRAM2	136	F	CTGTGCTTACCTTTGGTATGGG	22	61	166235181c1
		R	GCACTTACTCCACACCAGATAAC	23	61	
GAA	129	F	TGCCCTCGCAGTATATCACAG	21	61	119393892c3
		R	GAGACCCGTAGAGGTTCGC	19	62	
HSP90AA1	187	F	GCTTGACCAATGACTGGGAAG	21	61	153792589c3
		R	AGCTCCTCACAGTTATCCATGA	22	60	
IRGM	89	F	GCCATGAATGTTGAGAAAGCCT	22	61	224809575c1
		R	GTCCTGGACACTATCTTCAGAGT	23	61	
NPC1	189	F	GTCCAGCGCAGGTGTTTTC	19	62	255652943c1
		R	GCCGAACATCACAACAGAGAC	21	61	
SQSTM1	116	F	AAGCCGGGTGGGAATGTTG	19	63	214830450c3
		R	CCTGAACAGTTATCCGACTCCAT	23	61	
TNFα	91	F	GAGGCCAAGCCCTGGTATG	19	62	25952110c2
		R	CGGGCCGATTGATCTCAGC	19	63	
GAPDH	101	F	CTGGGCTACACTGAGCACC	19	62	378404907c3
		R	AAGTGGTCGTTGAGGGCAATG	21	63	

Supplementary Table S4: Human Autophagy PCR Array genes modulated upon SIRT6 knockdown in A375 melanoma cells.

Gene	Gene Description	Autophagy Machinery Components	Fold Change
AKT1	V-akt murine thymoma viral oncogene homolog 1	Co-Regulators of Autophagy and Apoptosis	-2
AMBRA1	Autophagy/beclin-1 regulator 1	Involved in Autophagic Vacuole Formation	1
APP	Amyloid beta (A4) precursor protein	Co-Regulators of Autophagy and Apoptosis	1
ATG10	ATG10 autophagy related 10 homolog	Responsible for Protein Transport	2.03
ATG12	ATG12 autophagy related 12 homolog	Involved in Autophagic Vacuole Formation, Co-Regulators of Autophagy and Apoptosis	-2
ATG16L1	ATG16 autophagy related 16-like 1	Involved in Autophagic Vacuole Formation, Responsible for Protein Transport	1.01
ATG16L2	ATG16 autophagy related 16-like 2	Responsible for Protein Transport	-1.59
ATG3	ATG3 autophagy related 3 homolog	Involved in Protein Ubiquitination, Responsible for Protein Transport	-2
ATG4A	ATG4 autophagy related 4 homolog A	Involved in Autophagic Vacuole Formation, Responsible for Protein Targeting to Membrane / Vacuole, Responsible for Protein Transport, Gene with Protease Activity	1
ATG4B	ATG4 autophagy related 4 homolog B	Involved in Autophagic Vacuole Formation, Responsible for Protein Targeting to Membrane / Vacuole, Responsible for Protein Transport, Gene with Protease Activity	1
ATG4C	ATG4 autophagy related 4 homolog C	Involved in Autophagic Vacuole Formation, Responsible for Protein Targeting to Membrane / Vacuole, Responsible for Protein Transport, Gene with Protease Activity	-1
ATG4D	ATG4 autophagy related 4 homolog D	Involved in Autophagic Vacuole Formation, Responsible for Protein Targeting to Membrane / Vacuole, Responsible for Protein Transport, Gene with Protease Activity	-1
ATG5	ATG5 autophagy related 5 homolog	Involved in Autophagic Vacuole Formation, Co-Regulators of Autophagy and Apoptosis	1
ATG7	ATG7 autophagy related 7 homolog	Responsible for Protein Transport, Involved in Protein Ubiquitination	-2
ATG9A	ATG9 autophagy related 9 homolog A	Involved in Autophagic Vacuole Formation, Responsible for Protein Transport	1
ATG9B	ATG9 autophagy related 9 homolog B	Involved in Autophagic Vacuole Formation	-1.26
BAD	BCL2-associated agonist of cell death	Co-Regulators of Autophagy and Apoptosis	1
BAK1	BCL2-antagonist/killer 1	Co-Regulators of Autophagy and Apoptosis	-2.01
BAX	BCL2-associated X protein	Co-Regulators of Autophagy, Apoptosis and the Cell Cycle	1
BCL2	B-cell CLL/lymphoma 2	Co-Regulators of Autophagy and Apoptosis	-1.58
BCL2L1	BCL2-like 1	Co-Regulators of Autophagy and Apoptosis	-2
BECN1	Beclin 1, autophagy related	Co-Regulators of Autophagy and Apoptosis, Involved in Autophagic Vacuole Formation	-1
BID	BH3 interacting domain death agonist	Co-Regulators of Autophagy and Apoptosis	-1.58
BNIP3	BCL2/adenovirus E1B 19kDa interacting protein 3	Co-Regulators of Autophagy and Apoptosis	-1.58
CASP3	Caspase 3, apoptosis-related cysteine peptidase	Co-Regulators of Autophagy and Apoptosis	1
CASP8	Caspase 8, apoptosis-related cysteine peptidase	Co-Regulators of Autophagy and Apoptosis	-1
CDKN1B	Cyclin-dependent kinase inhibitor 1B (p27, Kip1)	Co-Regulators of Autophagy, Apoptosis and the Cell Cycle	-1.6
CDKN2A	Cyclin-dependent kinase inhibitor 2A (melanoma, p16, inhibits CDK4)	Co-Regulators of Autophagy, Apoptosis and the Cell Cycle	-1
CLN3	Ceroid-lipofuscinosis, neuronal 3	Co-Regulators of Autophagy and Apoptosis	-2
CTSB	Cathepsin B	Co-Regulators of Autophagy and Apoptosis	-2.01
CTSD	Cathepsin D	Autophagy in Response to Other Intracellular Signals	-1.01
CTSS	Cathepsin S	Autophagy in Response to Other Intracellular Signals	-4
CXCR4	Chemokine (C-X-C motif) receptor 4	Co-Regulators of Autophagy and Apoptosis	1
DAPK1	Death-associated protein kinase 1	Co-Regulators of Autophagy and Apoptosis	1
DRAM1	DNA-damage regulated autophagy modulator 1	Co-Regulators of Autophagy and Apoptosis, Gene Linking Autophagosome to Lysosome	1
DRAM2	DNA-damage regulated autophagy modulator 2	Autophagy in Response to Other Intracellular Signals	-2
EIF2AK3	Eukaryotic translation initiation factor 2-alpha kinase 3	Co-Regulators of Autophagy and Apoptosis, Autophagy Induction by Intracellular Pathogens	1.01
EIF4G1	Eukaryotic translation initiation factor 4 gamma, 1	Autophagy in Response to Other Intracellular Signals	1
ESR1	Estrogen receptor 1	Autophagy in Response to Other Intracellular Signals	1
FADD	Fas (TNFRSF6)-associated via death domain	Co-Regulators of Autophagy and Apoptosis	-1.59
FAS	Fas (TNF receptor superfamily, member 6)	Co-Regulators of Autophagy and Apoptosis	-1
GAA	Glucosidase, alpha; acid	Autophagy in Response to Other Intracellular Signals	2.01
GABARAP	GABA(A) receptor-associated protein	Involved in Autophagic Vacuole Formation, Responsible for Protein Transport, Gene Linking Autophagosome to Lysosome	1

Continued from previous page

GABARAPL1	GABA(A) receptor-associated protein like 1	Involved in Autophagic Vacuole Formation, Responsible for Protein Transport	1
GABARAPL2	GABA(A) receptor-associated protein-like 2	Involved in Autophagic Vacuole Formation, Responsible for Protein Transport	-1.57
HDAC1	Histone deacetylase 1	Co-Regulators of Autophagy and Apoptosis	1
HDAC6	Histone deacetylase 6	Involved in Protein Ubiquitination	1
HGS	Hepatocyte growth factor-regulated tyrosine kinase substrate	Autophagy in Response to Other Intracellular Signals	1
HSP90AA1	Heat shock protein 90kDa alpha (cytosolic), class A member 1	Chaperone-Mediated Autophagy	-2.02
HSPA8	Heat shock 70kDa protein 8	Chaperone-Mediated Autophagy	-1
HTT	Huntingtin	Co-Regulators of Autophagy and Apoptosis	1
IFNG	Interferon, gamma	Co-Regulators of Autophagy, Apoptosis and the Cell Cycle, Autophagy Induction by Intracellular Pathogens	1
IGF1	Insulin-like growth factor 1 (somatomedin C)	Co-Regulators of Autophagy and Apoptosis	1
INS	Insulin	Co-Regulators of Autophagy and Apoptosis	1
IRGM	Immunity-related GTPase family, M	Involved in Autophagic Vacuole Formation	-3.22
LAMP1	Lysosomal-associated membrane protein 1	Gene Linking Autophagosome to Lysosome, Autophagy Induction by Intracellular Pathogens	1.01
MAP1LC3A	Microtubule-associated protein 1 light chain 3 alpha	Involved in Autophagic Vacuole Formation	1
MAP1LC3B	Microtubule-associated protein 1 light chain 3 beta	Involved in Autophagic Vacuole Formation	1.02
MAPK14	Mitogen-activated protein kinase 14	Autophagy in Response to Other Intracellular Signals	-1
MAPK8	Mitogen-activated protein kinase 8	Co-Regulators of Autophagy and Apoptosis	-1
MTOR	Mechanistic target of rapamycin (serine/threonine kinase)	Co-Regulators of Autophagy and Apoptosis	1
NFKB1	Nuclear factor of kappa light polypeptide gene enhancer in B-cells 1	Co-Regulators of Autophagy and Apoptosis	-1.26
NPC1	Niemann-Pick disease, type C1	Gene Linking Autophagosome to Lysosome	-2
PIK3C3	Phosphoinositide-3-kinase, class 3	Autophagy in Response to Other Intracellular Signals	1
PIK3CG	Phosphoinositide-3-kinase, catalytic, gamma polypeptide	Co-Regulators of Autophagy and Apoptosis	-1.04
PIK3R4	Phosphoinositide-3-kinase, regulatory subunit 4	Autophagy in Response to Other Intracellular Signals	1
PRKAA1	Protein kinase, AMP-activated, alpha 1 catalytic subunit	Co-Regulators of Autophagy and Apoptosis	1
PTEN	Phosphatase and tensin homolog	Co-Regulators of Autophagy, Apoptosis and the Cell Cycle	1
RAB24	RAB24, member RAS oncogene family	Responsible for Protein Transport	1
RB1	Retinoblastoma 1	Co-Regulators of Autophagy and the Cell Cycle	1
RGS19	Regulator of G-protein signaling 19	Involved in Autophagic Vacuole Formation	1
RPS6KB1	Ribosomal protein S6 kinase, 70kDa, polypeptide 1	Autophagy in Response to Other Intracellular Signals	-1
SNCA	Synuclein, alpha (non A4 component of amyloid precursor)	Co-Regulators of Autophagy and Apoptosis	1.28
SQSTM1	Sequestosome 1	Co-Regulators of Autophagy and Apoptosis	-2
TGFB1	Transforming growth factor, beta 1	Co-Regulators of Autophagy, Apoptosis and the Cell Cycle	1
TGM2	Transglutaminase 2 (C polypeptide, protein-glutamine-gamma-glutamyltransferase)	Co-Regulators of Autophagy and Apoptosis	1
TMEM74	Transmembrane protein 74	Autophagy in Response to Other Intracellular Signals	-1.59
TNF	Tumor necrosis factor	Co-Regulators of Autophagy and Apoptosis	-32.05
TNFSF10	Tumor necrosis factor (ligand) superfamily, member 10	Co-Regulators of Autophagy and Apoptosis	-1.03
TP53	Tumor protein p53	Co-Regulators of Autophagy, Apoptosis and the Cell Cycle	1
ULK1	Unc-51-like kinase 1 (C. elegans)	Involved in Autophagic Vacuole Formation	1
ULK2	Unc-51-like kinase 2 (C. elegans)	Autophagy in Response to Other Intracellular Signals	1
UVRAG	UV radiation resistance associated gene	Autophagy in Response to Other Intracellular Signals	1
WIPI1	WD repeat domain, phosphoinositide interacting 1	Involved in Autophagic Vacuole Formation	1
